# Monthly Report of Diseases

**Published:** 1800-09

**Authors:** 


### MONTHLY REPORT of DISEASES,

### Admitted under the Care of the PHYSICIANS of the FINSBURY DISPENSARY, St. John's Square, Clerkenwell.

*The District, in which the Patients of the Finsbury Dispensary are visited, comprehends the Parishes of St. James, and of St. John, Clerkenwell; of St. Luke; of St. Sepulchre within and without; of St. Bartholomew, the Great and the Less; the Liberties of the Rolls, and of Glass-House Yard; the Town of Islington; the Parishes of St. Pancras; of St. Andrew, Holborn; and of St. George the Martyr, Queen's-square. This Tract of Ground may properly enough be termed, a North Western District of the Metropolis.*

LIST of DISEASES, &c. from July 20, to August 20, 1800.

| | No. of Cases. | | No. of Cases. |
|---|---|---|---|
| Cholera and Diarrhœa - | 67 | Amenorrhœa - - - - | 15 |
| Dysenteria - - - - - - | 4 | Leucorrhœa - - - - - - | 6 |
| Colica Pictonum - - - - - | 2 | Rheumatism - - - - - - | 9 |
| Asthenia & Dyspepsia - | 17 | Nephralgia - - - - - - | 3 |
| Prolapsus Ani - - - - - | 1 | Catarrhus - - - - - - | 13 |
| Typhus - - - - - - - - | 19 | Pertussis - - - - - - - | 3 |
| Phthisis - - - - - - - - | 8 | Cyn. Tonsil. - - - - - | 5 |
| Menorrhagia - - - - - | 7 | Cephalæa - - - - - - | 4 |

Scrophula

| | | | |
|---|---|---|---|
| Schrophula | 8 | Vermes | 11 |
| Anafarca | 6 | Epiftaxis | 2 |
| Pfora | 1 | Epilepfia | 4 |
| Hydrocephalus | 3 | | |

The above lift will fhow, that the typhoid contagion ftill prevails in the Finfbury diftrict, although with a degree of virulence that is by no means equal to that which it exhibited during the preceding month. Rarely has there been a feafon in which this complaint has appeared fo decidedly epidemic, and fo troublefome and alarming in its fymptoms. The morbid affection of the brain was never, perhaps, a more uniform attendant of this fever, in confequence, it is highly probable, of the unufually intenfe and long-continued heat of the fummer. One circumftance denoting danger in Typhus, during hot weather, which has not hitherto been, at leaft publicly, noticed, is the fixing of flies upon the face, neck, and arms of the patient, a circumftance which may partly arife from his indifpofition or inability to drive them away; but ftill more perhaps from a tendency to a cadaverous ftate, as it is well known that thefe infects have a particular attachment for bodies which the vital principle has either deferted altogether, or on which its operation is in any confiderable degree diminifhed.

During this laft fortnight the fever, vulgarly called putrid, has been gradually difappearing, and Cholera, as is ufual in the autumnal period of the year, has become the popular diforder. This is a complaint which requires more vigilance on the part of a medical attendant than perhaps any other by which the human conftitution can be attacked, as it is not uncommon for it to carry off the patient, by its fatal violence, in the courfe of fo fhort a period as four and twenty hours.

W. W.
J. R.

*No. 63, Hatton Garden.*

